# Substoichiometric Silicon Nitride – An Anode Material for Li-ion Batteries Promising High Stability and High Capacity

**DOI:** 10.1038/s41598-018-26769-8

**Published:** 2018-06-05

**Authors:** Asbjørn Ulvestad, Hanne F. Andersen, Ingvild J. T. Jensen, Trygve T. Mongstad, Jan Petter Mæhlen, Øystein Prytz, Martin Kirkengen

**Affiliations:** 1 Department of Battery Technology, Institute for Energy Technology, P.O. Box 40, NO-2027 Kjeller, Norway; 2 Department of Physics, Centre for Materials Science and Nanotechnology, University of Oslo, P.O. Box 1048 Blindern, NO-0316 Oslo, Norway; 3 SINTEF Industry, P.O. Box 124 Blindern, NO-0314 Oslo, Norway

## Abstract

Silicon is often regarded as a likely candidate to replace graphite as the main active anode material in next-generation lithium ion batteries; however, a number of problems impacting its cycle stability have limited its commercial relevance. One approach to solving these issues involves the use of convertible silicon sub-oxides. In this work we have investigated amorphous silicon sub-nitride as an alternative convertible silicon compound by comparing the electrochemical performance of a-SiN_x_ thin films with compositions ranging from pure Si to SiN_0.89_. We have found that increasing the nitrogen content gradually reduces the reversible capacity of the material, but also drastically increases its cycling stability, e.g. 40 nm a-SiN_0.79_ thin films exhibited a stable capacity of more than 1,500 mAh/g for 2,000 cycles. Consequently, by controlling the nitrogen content, this material has the exceptional ability to be tuned to satisfy a large range of different requirements for capacity and stability.

## Introduction

Silicon has attracted much attention as a potential next generation anode material for lithium ion batteries (LIBs) due to its high theoretical specific capacity (3579 mAh/g (Li_15_Si_4_)) compared to that of the commonly used graphite (372 mAh/g)^1^. Despite much effort to develop silicon anodes, carbonaceous anodes are still preferred in the vast majority of commercial LIBs, because of a number of unresolved stability issues with silicon. The large volume change the material goes through during lithiation and delithiation results in internal stress build-up, causing the material to fracture and pulverize. In addition to exposing new surface area, the pulverization can also electrically disconnect material from the current collector, rendering it inactive. Numerous attempts have been made to circumvent these challenges by nano-structuring the silicon to reduce the internal stress formation^2^. Thin films^2^, nanoparticles^3^ and nanorods/wires^4^, in both compact^4^ and porous^3^ configurations, have been investigated with varying success^1,5,6^. The resulting large surface areas of nanomaterials have also called for investigations into surface modifications and coatings^4,7–9^.

A different approach has been to use compounds known as convertible oxides or *in-situ* formed alloy anode materials^10–12^. Fuji Photo Film Co. first reported on the tin based composite oxide electrode in 1997^10^, and the principle has since been extended to silicon sub-oxides^13–17^. These materials go through an irreversible conversion reaction during the first lithiation cycle, in which they form a fine dispersion of active metal particles in an inactive oxide matrix^18^. The function of this matrix is to provide a buffer for the large volume change of the silicon domains during lithiation and delithiation, while also maintaining sufficient lithium ion conductivity in the material. The nature of convertible electrode materials is thus a trade-off where cycling stability is gained at the expense of lowering the reversible capacity and increasing the first cycle irreversible capacity. Convertible materials are not to be confused with conversion electrode materials, whose conversion reaction is reversible, and is in itself the source of the reversible capacity^19^.

There have also been several reports on the reversible lithiation of different ternary nitrides, e.g. Li_3-x_FeN_2_ (0 < x < 1.0)^20^ and Li_7-x_MnN_4_(0 < x < 1.25)^21^. These are, however, not convertible materials, but are believed to function by a reversible reconstitution reaction^18^, and have theoretical capacities of 256 and 210 mAh/g, respectively. Several binary nitrides are also known to undergo electrochemical reactions with lithium. Nanocrystalline M_3_N (M = Co^22^, Fe^22^, Ni^23^) is believed to form M and Li_3_N during the initial lithiation, with the capacity in the subsequent cycles relying on the partial decomposition of Li_3_N and nitridation of the transition metal, and vice versa. Sn_3_N_4_^24^, Ge_3_N_4_^25^ and silicon tin oxynitrides^26^, on the other hand, are believed to function more along the lines of the convertible oxides in that an irreversible conversion to metallic Sn/Ge/Si and Li_3_N is followed by a subsequent reversible lithiation and delithiation of the metal particles^27^. Zn_3_N_2_ was previously grouped with these materials^24^, but have later been shown to function as an intermediate between a convertible electrode and a conversion electrode material^28^.

Only a small number of reports have been made on the use of silicon nitride in lithium ion batteries, and these have reported varying results for different material forms and compositions. In the context of lithium ion batteries, stoichiometric silicon nitride has been treated both as an inactive material, used as an inactive scaffolding material for silicon based anodes^29^, as well as an active material^30^, albeit with a very low capacity of only 40 mAh/g. Non-stoichiometric SiN_x_, on the other hand, have been shown to exhibit far higher capacities: Ahn, *et al*.^31^ have reported on the performance of SiN_0.32_ and SiN_0.69_ thin film electrodes, the former exhibiting negligible capacity before experiencing a sudden increase in capacity to approximately 2,300 mAh/g after several hundred cycles, while the latter stayed below 80 mAh/g for the entire duration of the cycling. Suzuki, *et al*.^32^ have reported that 200 nm and 500 nm SiN_0.92_ thin films exhibited first cycle reversible capacities of approximately 1,500 mAh/g and 1,000 mAh/g, and retained capacities of 1,300 mAh/g and 700 mAh/g after 100 cycles, respectively. Similar thin films of a-SiN_0.89_ have also been found to exhibit a comparable reversible capacity of more than 1250 mAh/g^33^ over 2400 cycles. In these works, silicon nitride is assumed to function as a conversion material, forming active silicon and an inactive nitride matrix component^31–33^. The exact nature of the matrix has not yet been determined, but it has been suggested to consist of Si_3_N_4_ and/or Li_3_N^30,31^, or a ternary lithium silicon nitride, e.g. Li_2_SiN_2_^32,33^. The following reaction schematically describes the general processes of conversion of SiN_x_ and subsequent cycling:

$${\boldsymbol{S}}{\boldsymbol{i}}{{\boldsymbol{N}}}_{{\boldsymbol{x}}}+{\boldsymbol{n}}{\boldsymbol{L}}{{\boldsymbol{i}}}^{+}+{{\boldsymbol{n}}{\boldsymbol{e}}}^{-}\mathop{\to }\limits^{{\boldsymbol{C}}{\boldsymbol{o}}{\boldsymbol{n}}{\boldsymbol{v}}{\boldsymbol{e}}{\boldsymbol{r}}{\boldsymbol{s}}{\boldsymbol{i}}{\boldsymbol{o}}{\boldsymbol{n}}}{\boldsymbol{M}}{\boldsymbol{a}}{\boldsymbol{t}}{\boldsymbol{r}}{\boldsymbol{i}}{\boldsymbol{x}}+{\boldsymbol{L}}{{\boldsymbol{i}}}_{3.75}{\boldsymbol{S}}{\boldsymbol{i}}\mathop{\rightleftarrows }\limits^{\,{\boldsymbol{C}}{\boldsymbol{y}}{\boldsymbol{c}}{\boldsymbol{l}}{\boldsymbol{i}}{\boldsymbol{n}}{\boldsymbol{g}}\,}{\boldsymbol{M}}{\boldsymbol{a}}{\boldsymbol{t}}{\boldsymbol{r}}{\boldsymbol{i}}{\boldsymbol{x}}+{\boldsymbol{S}}{\boldsymbol{i}}+{\bf{3.75}}{\boldsymbol{L}}{{\boldsymbol{i}}}^{+}+{{\bf{3.75}}{\boldsymbol{e}}}^{-}$$

Similarly as for the silicon sub-oxides, the expected function of the matrix is primarily to buffer the volume change of the active silicon domains during lithiation and delithiation, while providing necessary lithium ion conductivity for lithium to reach these domains. As the nitrogen inhibits the reversible lithiation and delithiation of the silicon bound in the matrix, the material can be regarded as a self-limiting silicon electrode, effectively reducing the volume change during cycling at the cost of some reversible capacity.

In non-stoichiometric SiN_x_, the addition of nitrogen is expected to increase cycle stability, but reduce the reversible capacity and increase the irreversible capacity; however, given the span of experimental conditions used in previous works, a comparison is difficult to make. In this work we have therefore aimed to investigate a large number of different electrodes under comparable conditions, with both a range of different compositions and different thicknesses, in order to determine how these parameters affect the capacity and cycling performance of the material. The results show that, while the addition of nitrogen does indeed gradually reduce the reversible capacity of the material, as well as increase the first cycle irreversible capacity, even the most nitrogen rich films, SiN_0.89_, still had a reversible capacity of approximately 1,200 mAh/g. The best performing electrodes in this work were 40 nm a-SiN_0.79_ thin films, which exhibited a stable reversible capacity of more than 1,500 mAh/g for 2,000 cycles.

## Results and Discussion

### Film characterization

**Figure 1:**
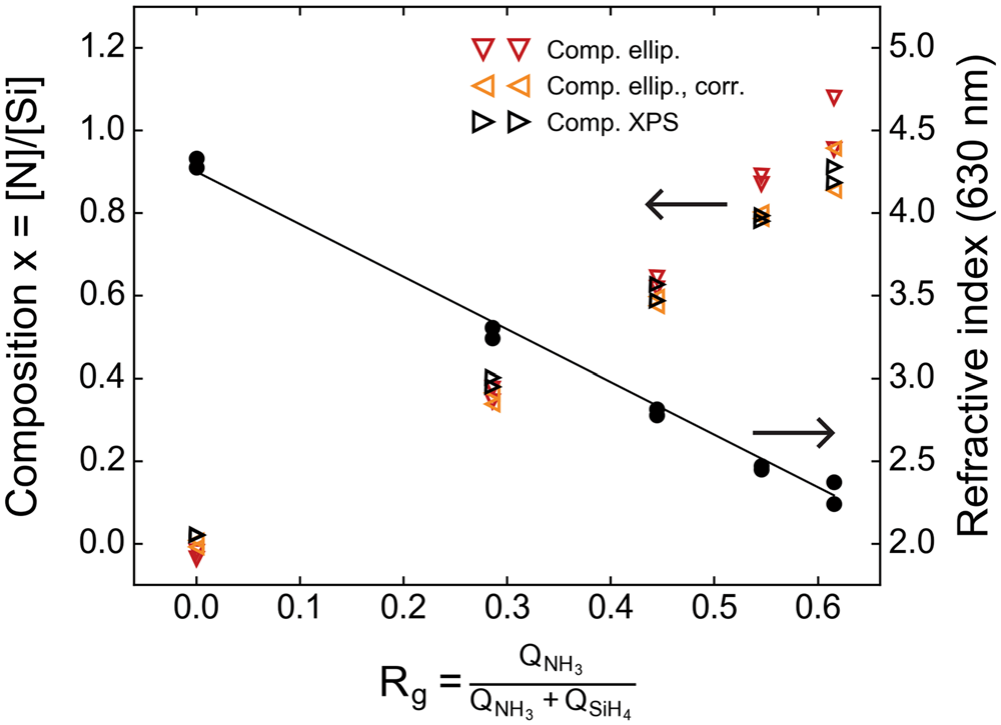
Refractive index and composition of the films as a function of plasma composition, R_g_. Refractive indices are measured by ellipsometry, and the compositions are both measured by XPS and estimated from the refractive index.

For the experiments in this work, thin films were deposited directly on copper foil current collectors using plasma enhanced chemical vapour deposition (PECVD) with silane (SiH_4_) and ammonia (NH_3_) as precursors. By varying precursor flow rates and deposition times, films were made with five different compositions (A-E) and two thicknesses of each composition (1-2). In order to provide the necessary grounds for interpretation of data from the electrochemical analyses, a thorough characterization of the pristine films was conducted. An overview of the properties of the as-deposited films can be seen in Table 1, as measured by ellipsometry, X-ray photoelectron spectroscopy (XPS), and transmission electron microscopy (TEM). The somewhat higher thickness of films B1 and B2 is expected to be reflected in an accelerated degradation during cycling, as will be addressed later. The refractive indices measured using ellipsometry can also be seen in Fig. 1, plotted versus the PECVD plasma composition *R*_*g*_ = *Q*_*NH*3_*/(Q*_*NH*3_ + *Q*_*SiH*4_). An approximately linear trend is observed, decaying from *n* = 4.2 at *R*_*g*_ = 0, which is consistent with the refractive index of pure amorphous silicon. This figure also shows the compositions measured by XPS and estimated from the refractive indices using a relation proposed by Bustarret, *et al*.^34^ (Eq. 1 in Methods). While similar, particularly at lower R_g_, the trend shows that ellipsometry overestimates the nitrogen content compared to XPS. The optical properties of the films are somewhat dependent on the hydrogen content, as well as the porosity, and therefore also on the deposition parameters. Since the compositions are calculated from the refractive indices using an empirical expression with reference indices from literature, discrepancies may occur; hence we take the values obtained from XPS to be more credible. Given that both films made using the same process parameters should ideally have the same composition, the average of the values measured in XPS will be used in the remainder of this paper in order to reduce the effect of experimental variations. As a side note: Eq. 1 can be adapted by fitting it to the XPS measured compositions, using ${n}_{S{i}_{3}{N}_{4}}$ as the fitting parameter and *n*_*Si*_ equal to the refractive index measured for the pure silicon film (*n*_*Si*_ = 4.30). This fitting resulted in ${n}_{Si{N}_{1.31}}=1.84$, and the compositions seen in Table 1 and Fig. 1.

**Table 1:** Film parameters as measured by ellipsometry, TEM and XPS.

Film index	Thickness [nm]	Refractive index	Composition, x = [N]/[Si]					Mass density, *ρ* [g/cm^3^]	
	Measured, ellipsometry	Measured, TEM	Ellipsometry n (λ = 630 nm)	Ellipsometry^a^	Ellipsometry,corr.^b^	XPS	XPS, avg.	***ρ***_***l***_^c^	***ρ***_***p***_^d^
A1	42.3 ± 0.8		4.28	−0.02	0.01	0.02	0.02 ± 0.00*	2.21	2.17
A2	124.1 ± 0.9		4.33	−0.04	−0.01	0.02			
B1	55.9 ± 0.1		3.31	0.35	0.34	0.38	0.39 ± 0.01	2.41	2.15
B2	176.6 ± 0.5	180	3.24	0.38	0.37	0.40			
C1	46.5 ± 0.1		2.78	0.64	0.60	0.59	0.61 ± 0.02	2.52	2.25
C2	127.5 ± 0.3	130	2.82	0.62	0.58	0.63			
D1	40.0 ± 0.1		2.47	0.87	0.79	0.79	0.79 ± 0.01	2.62	2.31
D2	133.6 ± 1.1	135	2.45	0.89	0.80	0.78			
E1	40.9 ± 0.4		2.24	1.08	0.96	0.91	0.89 ± 0.02	2.68	2.44
E2	114.2 ± 0.1	115	2.37	0.96	0.86	0.87			

^a^Calculated using equation 1 with *n*_*Si*_ = 4.21 and ${n}_{S{i}_{3}{N}_{4}}=2.01$.

^b^Calculated using equation 1 with *n*_*Si*_ = 4.30 and ${n}_{S{i}_{3}{N}_{4}}=1.84$.

^c^Calculated using equation 6 with the average compositions determined from XPS.

^d^Calculated using the plasmon energy measured using STEM-EELS and equations 2 to 5.

^*^Given that film A1 and A2 were deposited without ammonia, the small fraction of nitrogen measured in XPS is attributed to experimental error, and these film are regarded as pure silicon.

Measurements of the bulk plasmon energy of the materials were conducted on the 120 nm film of each composition using electron energy loss spectroscopy (EELS) in the TEM. The film mass densities were then determined from the bulk plasmon energy as described in the Methods section (Eqs 2 to 5), and were found to generally be increasing slightly with nitrogen content. This is as expected, but less pronounced than approximated by linear interpolation based on the densities of a-Si and a-SiN_1.31_ found in literature (Eq. 6 in Methods), as seen in Table 1. Given the wide range of SiN_x_ densities which have been reported for PECVD deposited a-SiN_x_, the values determined from the plasmon energies are regarded as more reliable in this instance. We confirmed this by conducting the same measurements and analysis on pure crystalline Si and Si_3_N_4_ (Sigma Aldrich), which resulted in densities of 2.38 g/cm^3^ and 3.24 g/cm^3^, respectively. These values correspond well with the expected densities at 2.33 g/cm^3^ and 3.2 g/cm^3^ (β-Si_3_N_4_)^35^. The band gaps used in Eq. 6 were 1.12 eV for c-Si^36^, 5.0 for c-Si_3_N_4_^37^ and otherwise interpolated from the measurements by Guraya, *et al*.^38^ for a-SiN_x_. Further details of this analysis can be found in the supplementary information.

**Figure 2:**
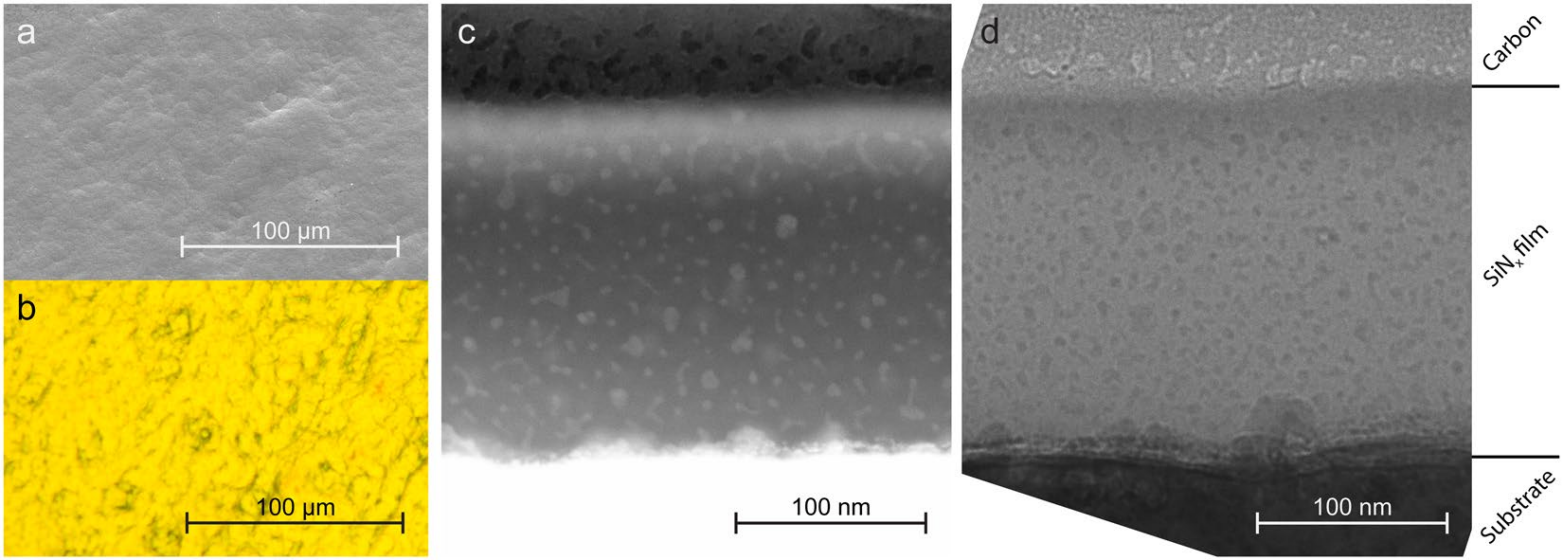
Optical, SEM and TEM micrographs of SiN_x_ thin films. SEM (**a**) and optical (**b**) micrographs of the surface of the 114 nm SiN_0.89_ film, showing an even film coverage, despite the relative roughness of the underlying copper substrates. STEM-HAADF (**c**) and TEM bright field (**d**) micrographs of the cross section of the 177 nm thick SiN_0.39_ film, showing signs of nanoscale segregation. The carbon coating seen in the top of the TEM images was added as a protective layer during focused ion beam (FIB) sample preparation for TEM.

Rolled copper foil was used as substrates for this work due to its availability and widespread use in battery fabrication. Such substrates are expected to have some surface roughness, which was confirmed in the SEM and optical microscopy, seen in Fig. 2a and b, respectively. It was, however, also determined that the surface coverage of the films was even, with no sign of island formation. The colour of the films in the optical micrographs is related to the thickness of the films, and the uniform colour indicates a relatively uniform thickness over the range observed in the images. These observations were corroborated by cross section analysis in the TEM (Fig. 2c,d), which showed that the films were deposited uniformly, despite the relative roughness of the underlying copper. The thickness measurements made in the TEM images were also consistent with those made by ellipsometry, as seen in Table 1.

**Figure 3:**
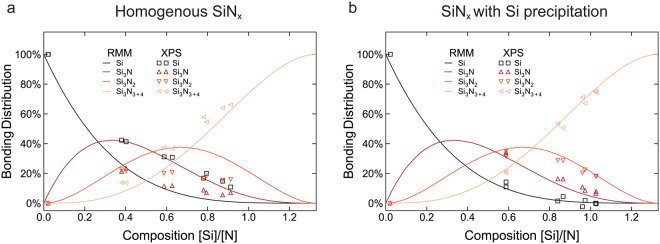
Distribution of silicon atoms in Si-Si_3_N, Si-Si_3_N_2_, Si-Si_3_N_3+4_ coordination states. Coordination distributions determined by peak fitting of the Si 2p peak in XPS spectra obtained from the different a-SiN_x_ thin films (markers), compared to the distribution predicted by a random mixing model (lines). (**a**) Assuming homogenous a-SiN_x_ and (**b**) assuming some pure silicon has precipitated.

The high resolution XPS spectra from the Si 2p region were fitted with Voigt functions in order to investigate the bonding state of silicon and nitrogen. The Si 2p peaks in the spectra from the pure silicon films, at 99.2 eV, were used to determine the shape of the silicon component (Si-Si_4_ tetrahedra). Components corresponding to Si-Si_3_N, Si-Si_3_N_2_, Si-SiN_3_, and Si-Si_3_N_4_ tetrahedra were added after a procedure from Ingo, *et al*.^39^. A fixed chemical shift of 0.62 eV between the components and an increase of the full width at half maximum of 0.2 eV for increasing N content in the tetrahedron were found to give consistent results for all samples, with peak positions consistent with those reported by Hayakawa, *et al*.^40^. This fitting performed on the spectra obtained from the different a-SiN_x_ thin films can be found in the supplementary information. A comparison of the resulting component distributions with the distribution derived from a random mixing model (RMM)^41^ can be seen in Fig. 3a, where the contributions from Si-SiN_3_ and Si-Si_3_N_4_ have been combined in an effort to prevent overfitting and reduce reliance on accurate separation of these wider peaks. This shows that the fraction of silicon atoms in a Si-Si_4_ configuration is higher in all the nitrides than the RMM predicts, which indicates that some short range ordering or phase separation has occurred in the films. Formation of nanoscale silicon clusters in silicon rich a-SiN_x_ films has previously been reported by other groups^42–44^. If assuming that a fraction of the material is indeed pure silicon, while the remaining a-SiN_x_ is correspondingly nitrogen enriched, the coordination distribution of the enriched a-SiN_x_ can be estimated by subtracting some pure silicon from the distribution determined from XPS. Using the amount of separated pure silicon in each film as fitting parameters, together with a recalculated composition of the enriched a-SiN_x,_ the resulting coordination distributions were fitted to those predicted by an RMM. The result can be seen in Fig. 3b, which shows that the distribution of coordination states in the enriched a-SiN_x_ is in good agreement with an RMM. Examination of the films with scanning transmission electron microscopy (STEM) and TEM confirmed that they do indeed contain clusters with diameters up to a few nanometres, as can be seen in Fig. 2c,d. While this pure silicon is expected to provide some lithiation capacity, the limited amount is only able to account for a small fraction of the measured capacity of the material; hence the a-SiN_x_ must necessarily play a dominant role in the observed reversible reactions with lithium.

### Electrochemical analysis

The electrochemical performance of the films was tested by galvanostatic cycling in half-cells against lithium metal counter electrodes, the details of which are found in the Methods section and supplementary information. Table 2 shows the average first cycle discharge and charge capacities, and Coulombic efficiency of several electrodes of each film. Note that all specific quantities are calculated based on the total mass of the SiN_x_ thin films. These results show that the reversible capacity of the material gradually decreases with increasing nitrogen content, as was expected. The first cycle Coulombic efficiency is also decreasing with increasing nitrogen content, which stems from the loss of lithium in the formation of the matrix phase. Due to the influence of surface losses, the Coulombic efficiency and irreversible capacity of films with different thicknesses cannot be directly compared; however, it is clear that increasing nitrogen content increases the bulk irreversible capacity.

**Table 2:** Overview of data from electrochemical testing of the different a-SiN_x_ thin films.

Film	Composition	1^st^ Cycle			Average over 2000 cycles		
		Specific Discharge Capacity [mAh/g]	Specific Charge Capacity [mAh/g]	Coulombic Efficiency [%]	Specific Discharge Capacity [mAh/g]	Specific Charge Capacity [mAh/g]	Coulombic Efficiency [%]
A1	Si	4,831 ± 176	3,029 ± 175	62.6 ± 1.5	1,240 ± 99	1,234 ± 98	>99%
B1	SiN_0.39_	4,186 ± 49	2,511 ± 57	60.0 ± 1.6	1,232 ± 11	1,226 ± 11	>99%
C1*	SiN_0.61_	4,046 ± 96	1,976 ± 26	48.8 ± 0.5	1,302 ± 53	1,296 ± 52	>99%
D1	SiN_0.79_	4,173 ± 30	1,689 ± 2	40.5 ± 0.3	1,574 ± 38	1,568 ± 37	>99%
E1	SiN_0.89_	3,593 ± 76	1,158 ± 8	32.2 ± 0.8	1,167 ± 12	1,161 ± 11	>99%
A2	Si	4,082 ± 132	3,410 ± 113	83.5 ± 1.2	1,266 ± 53	1,263 ± 54	>99%
B2	SiN_0.39_	3,311 ± 89	2,496 ± 100	75.4 ± 1.0	1,172 ± 28	1,170 ± 30	>99%
C2	SiN_0.61_	3,320 ± 25	2,208 ± 34	66.5 ± 0.5	1,226 ± 6	1,223 ± 4	>99%
D2	SiN_0.79_	2,516 ± 46	1,456 ± 26	57.9 ± 0.2	1,065 ± 17	1,068 ± 18	>99%
E2*	SiN_0.89_	2,385 ± 17	1,208 ± 13	50.7 ± 0.2	1,128 ± 22	1,134 ± 29	>99%

Average 1^st^ cycle specific discharge capacity, charge capacity and coulombic efficiency calculated from three*electrodes of each film, and the average specific discharge capacity, charge capacity and coulombic efficiency over the first 2000 cycles. The specific capacities are based on the total mass of the SiN_x_ films, and the ranges show the standard deviation between the different cells of each thin film. *For films C1 and E2 the average was calculated from two rather than three cells.

**Figure 4:**
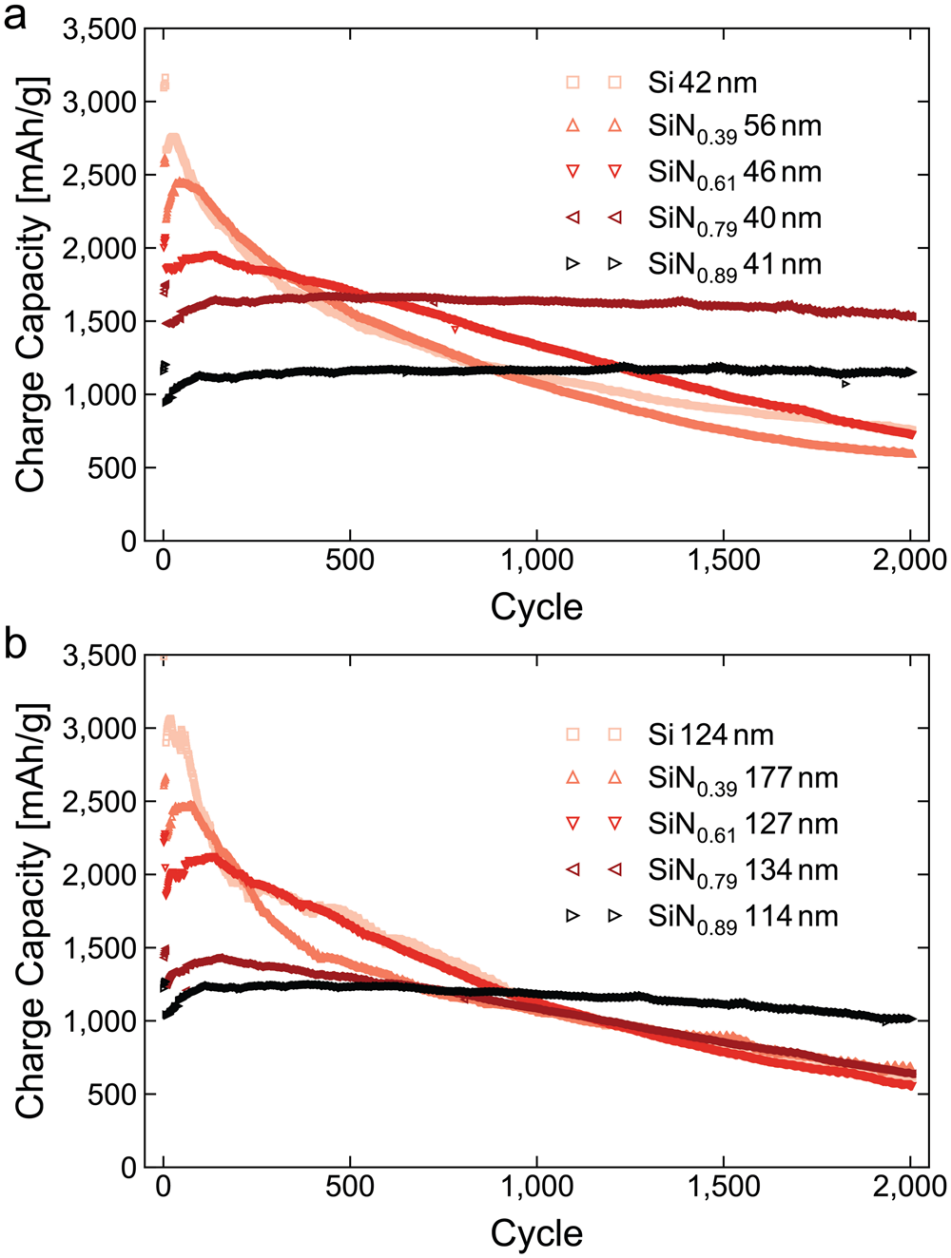
Cycle stability of a-SiN_x_ electrodes. Charge capacity during cycling for the thin (**a**) and thick (**b**) electrodes of each composition cycled for 2000 cycles at 1C after 6 formation cycles at C/20.

Figure 4 shows the development of the charge capacity (i.e. delithiation capacity in half-cell configuration) of electrodes of each different composition and thickness as they are cycled over 2,000 cycles. For the electrodes with the lowest nitrogen content (SiN_0.39_) there is no substantial cycle stabilization from the nitrogen. In the long term, an even more severe degradation of the SiN_0.39_ films is observed, as expected given the relatively higher thickness of these films. As the nitrogen content increases to SiN_0.61_, the capacity degradation becomes less pronounced, particularly in that there is no rapid drop during the first few hundred cycles, as is observed for the pure silicon electrode. For the thin electrodes, the stabilization comes into full effect for the two most nitrogen rich films, SiN_0.79_ and SiN_0.89_, which both exhibit excellent cycling stability, with practically no capacity loss over 2,000 cycles. For the thicker films more nitrogen is needed to stabilize the electrode, but the most nitrogen rich electrode, SiN_0.89_, reaches 2,000 cycles with only minor capacity degradation (97% of starting capacity and 81% of peak capacity). This correlates well with thicker films typically being more prone to internal stress build-up, and therefore require a lower content of active silicon relative to the volume buffering matrix to be properly stabilized. It is worth noting that others have reported better performance of pure silicon thin films than seen here^45^, which may be explained by poor adhesion to the copper substrate. Some interface impurities, mainly zinc, oxygen and phosphorous, were indeed observed using energy dispersive spectroscopy (EDS) in STEM, which may affect the film adhesion. It is, however, not expected to affect the validity of the comparison between the films.

**Figure 5:**
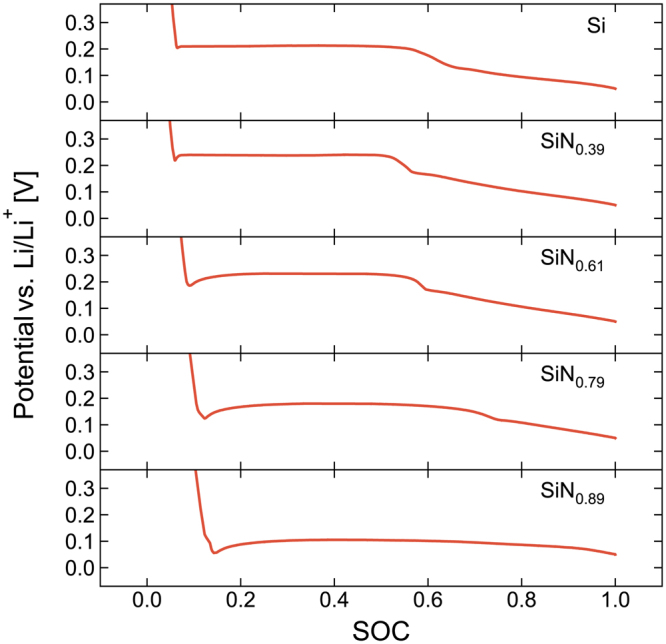
Voltage-State of Charge (SOC) plots of a-SiN_x_ electrodes. Voltage profiles focused on the initial lithiation plateau of the first cycle of the thicker film of each composition. Note the gradual change from the characteristic silicon two-step lithiation to a single curved plateau with increasing nitrogen content, as well as the development of a voltage minimum at the start of this plateau.

Figure 5 shows the voltage-capacity (VC) curves of the first lithiation of each of the thicker films, focused on the primary lithiation plateaus, during which the conversion reaction occurs. Evident from this figure is the gradual transformation from the typical silicon two stage lithiation to a single curved plateau as the nitrogen content increases. This figure also shows the development of a voltage minimum at the very start of the lithiation plateau. This overpotential is characteristic of a conversion reaction, and reflects the activation energy required for the nucleation of the new phases before the conversion can continue. As the nitrogen content increases, the overpotential becomes increasingly negative and eventually approaches the cut-off voltage. Using low current rates in the initial formation cycles may therefore be necessary for this material, depending on electrode type and nitrogen content. Too high current rate during conversion can potentially push the cell to the cut-off voltage prematurely. This is a possible explanation for the delayed start-up and limited capacity of silicon nitride reported by Ahn, *et al*.^31^ and Martín-Gil, *et al*.^30^, respectively.

**Figure 6:**
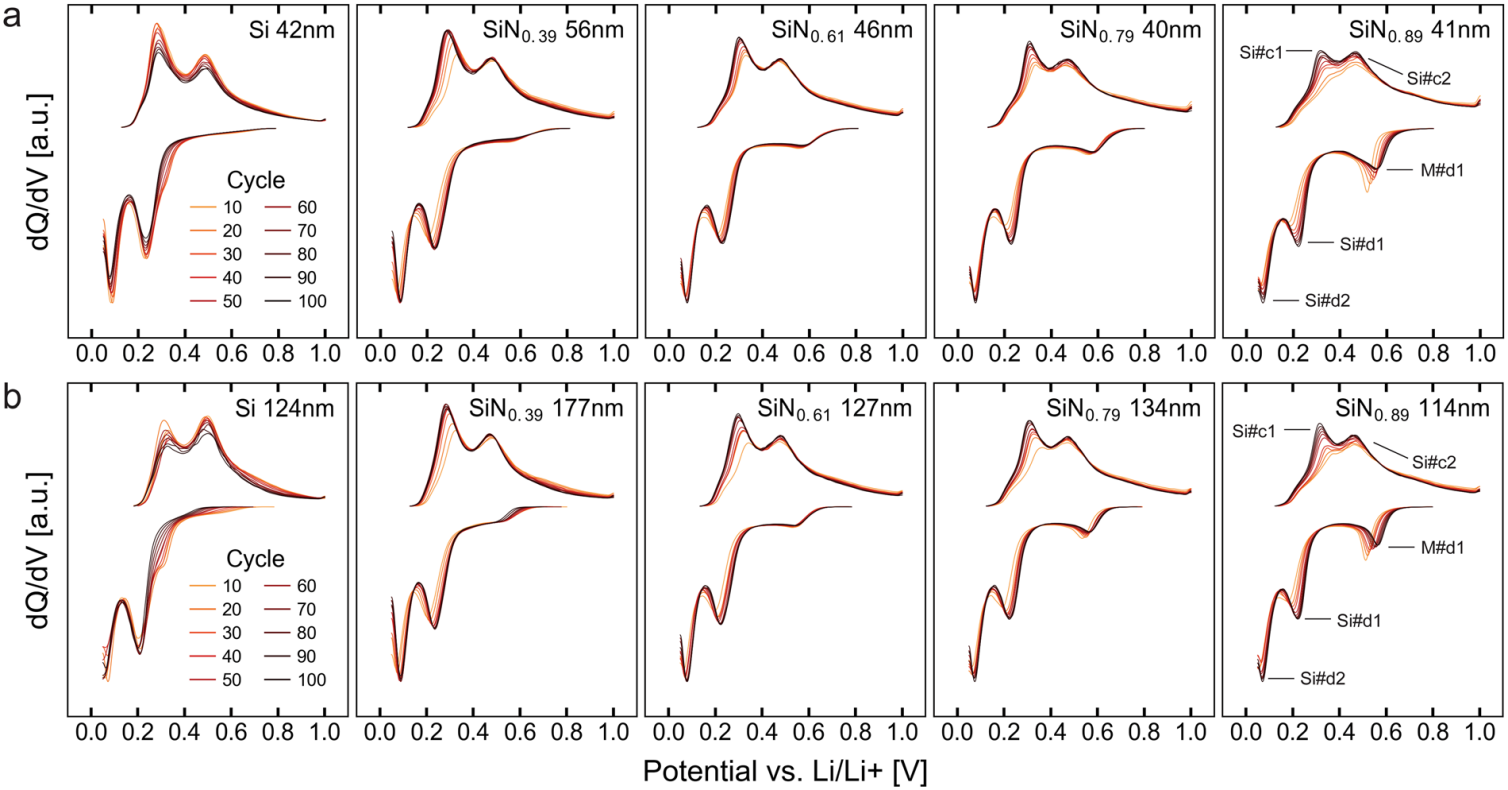
Differential capacity analysis of cycles 10–100. Plots of the differential capacity analysis calculated for every tenth cycle from cycles 10 to 100 of the different a-SiN_x_ electrodes, with increasing thickness from the top down and increasing nitrogen content from left to right.

Ogata, *et al*.^46^ have previously shown that lithiation of amorphous silicon happens mainly in two main stages occurring at ~250–300 mV and ~100 mV, the former corresponding to the gradual lithiation of the a-Si lattice to a composition of approximately Li_2.0_Si and the latter corresponding to the formation of a-Li_~3.5_Si from a-Li_2.0_Si^46–49^, hereinafter referred to as Si#d1 and Si#d2, respectively. Further lithiation eventually results in the formation of crystalline c-Li_15_Si_4_; however, this is largely avoided by using a cut-off at 50 mV^46^. During delithiation, the two processes happen in reverse, occurring at approximately 300 mV and 500 mV for a-Li_3.5_Si - > a-Li_2.0_Si and a-Li_2.0_Si - > a-Si, respectively, hereinafter referred to as Si#c1 and Si#c2. Figure 6a and b show the differential capacity analysis of cycles 10–100 for the different electrodes. These figures show that, in the cycles following the initial conversion reaction, the nitride electrodes do indeed begin to gradually exhibit a silicon-like electrochemical behaviour, with two lithiation peaks at 230 and 80 mV and corresponding delithiation peaks at 300 and 470 mV. Additionally, some high voltage activity is observed during delithiation of the nitrogen containing electrodes, manifesting as a very broad peak centred around 750 mV for the most nitrogen rich films. This does not occur in the pure silicon, and is therefore attributed to the partial delithiation of the matrix phase. This delithiation activity is assumed to be related to the additional lithiation peak at ~550 mV, M#d1, which is also only seen in the nitride films, and is therefore attributed to the “relithiation” of the matrix. It is also noted that these effects gradually increase in magnitude with increasing nitrogen content, while the peak positions remain constant. This supports the hypotheses that there is a phase segregation during conversion, and that, while higher nitrogen content increases the *amount* of matrix phase, its *composition* remains the same.

**Figure 7:**
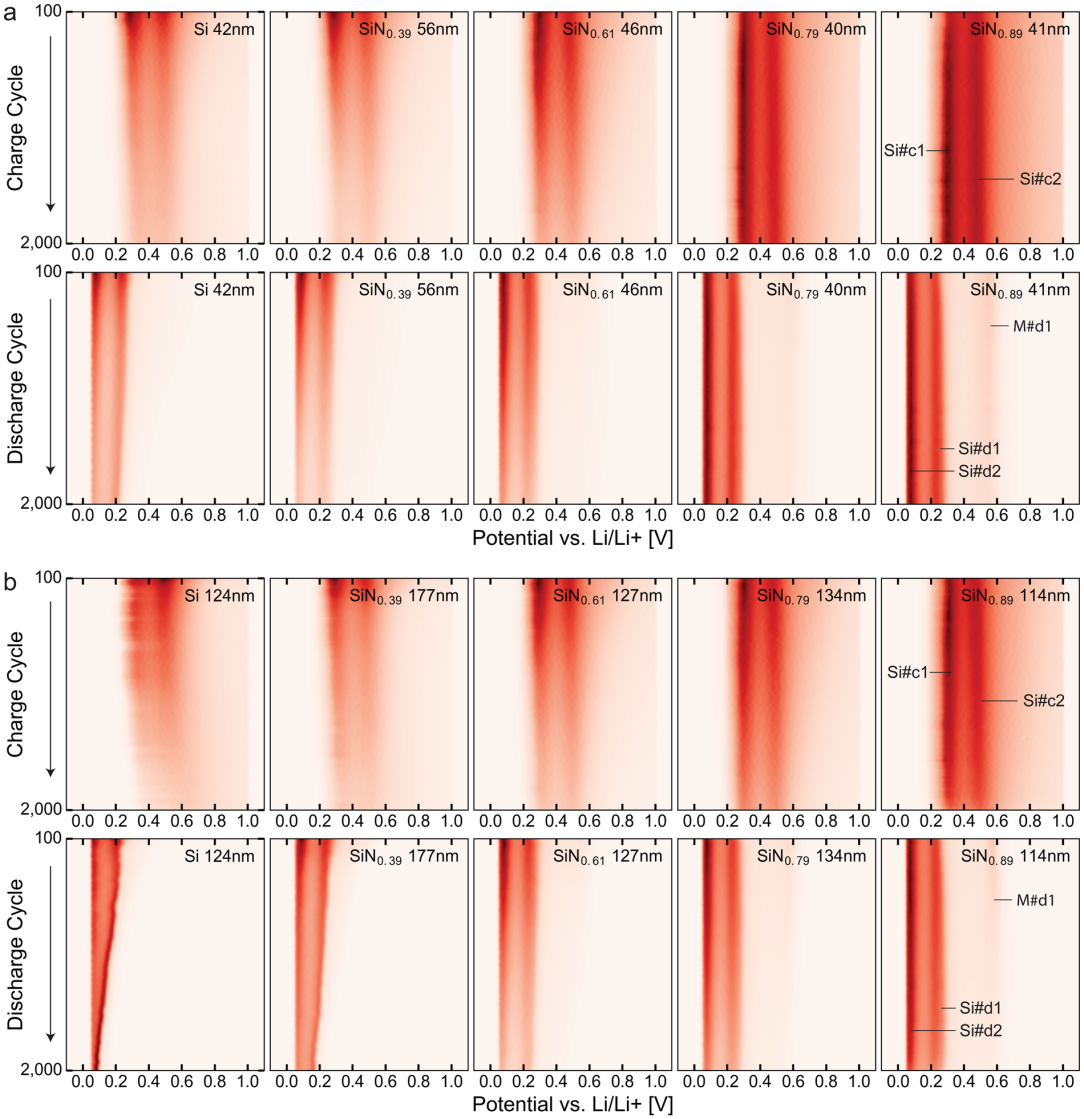
Differential capacity analysis of cycles 100–2,000. Colour maps showing the differential capacity analysis of the lithiation (discharge) and delithiation (charge) of the thin (**a**) and thick (**b**) electrodes of different compositions, showing the peak positions and intensities as they develop during cycles 100 to 2,000.

Figure 7a and b show the differential capacity analysis of the cells as it develops during long term cycling up to 2,000 cycles. While the cycling performance primarily experienced an improvement at higher nitrogen contents, this analysis reveals that there are significant differences in the workings of the electrodes already at lower nitrogen contents. It is clear from these figures that there are at least two different modes of degradation: one which primarily results in a decrease in peak magnitude and one which also results in a peak shift. Delamination is expected to deactivate parts of the electrode, while not significantly affecting the remaining active material, thus primarily causing a peak magnitude reduction. Pulverization of the film and subsequent formation of new SEI on the fresh underlying surface is expected to both deactivate material, as well as interfere with the lithium transport to the remaining active material, thus causing both a magnitude reduction and a peak shift. The thinner films are expected to be below the critical threshold thickness for internal fracturing/pulverization; hence the primary mode of degradation should be delamination. This is in agreement with the experiments, where capacity degradation is observed to be primarily by peak magnitude reduction. For the thicker electrodes, the pure silicon electrode experiences a considerable peak shift, indicating that the film is pulverized. This peak shift is reduced already for a-SiN_0.39_, and essentially absent for the more nitrogen rich films. These observations indicate that the improved cycling stability at higher nitrogen contents is largely related to the stabilization of the substrate-film interface; but more importantly, that the material itself is stabilized at lower nitrogen contents. This is not entirely unexpected, as the interface is primarily stabilized by a reduced volume change during cycling, while the material itself is expected to also be stabilized by increased lithium diffusion in the material and reduced silicon mobility. This has implications for the determination of a suitable composition of a-SiN_x_ in particle based electrodes, which are less dependent on interface stabilization than thin film electrodes.

**Figure 8:**
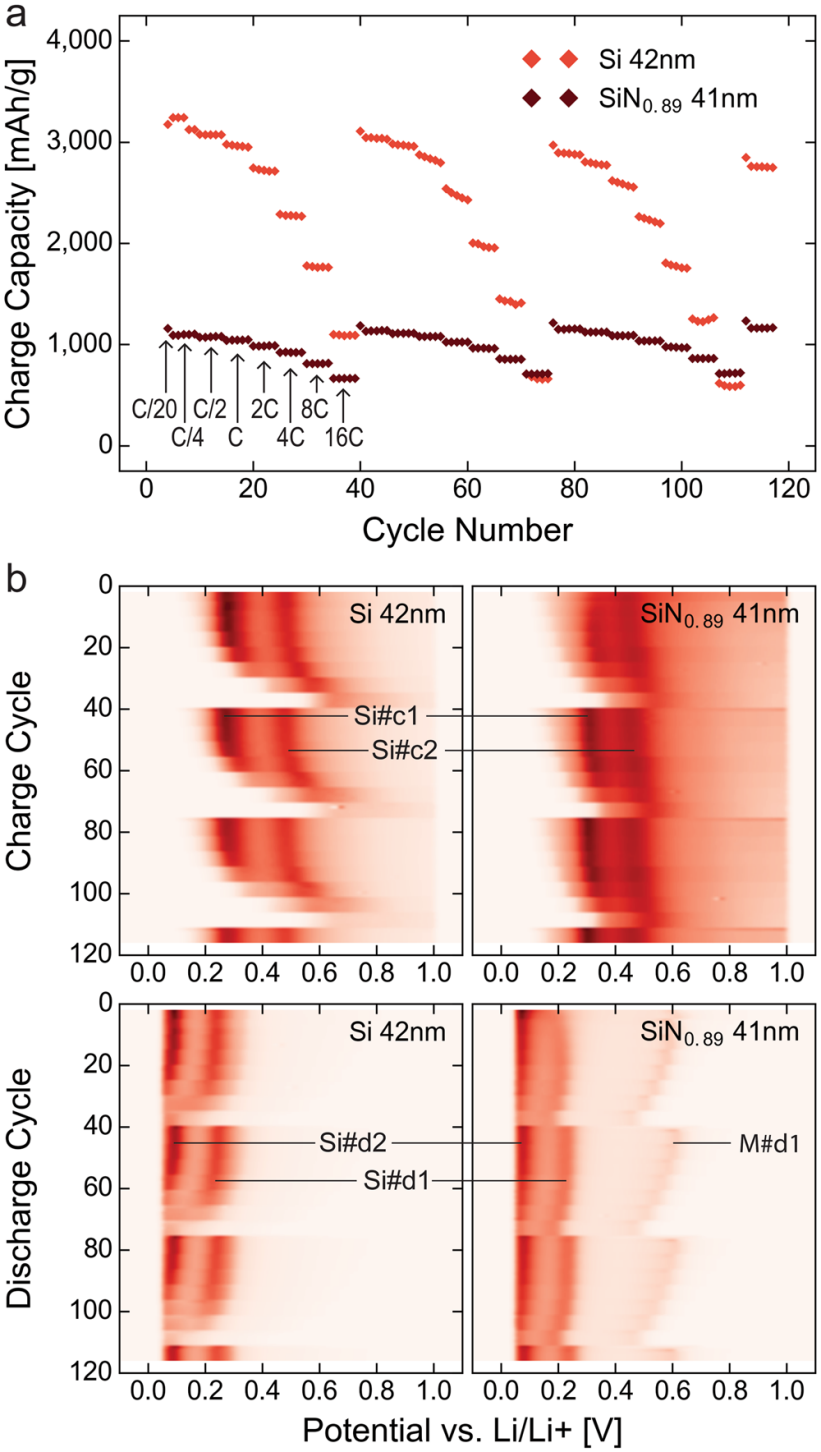
Rate test comparison of a 41 nm a-SiN_0.89_ electrode and a 42 nm pure silicon reference. (**a**) Charge capacity of a 42 nm Si and a 41 nm a-SiN_0.89_ electrode during rate testing from C/4 to 16C. (**b**) Colour maps of the differential capacity analysis of the same cells and cycles during charge and discharge, showing the shift in peak position with current rate.

Rate performance testing was conducted on electrodes made from the 42 nm Si and 41 nm SiN_0.89_ films. After a formation consisting of 3 cycles at a rate of C/20, these electrodes were cycled with a rate starting at C/4 which was doubled every 5 cycles up to 16C. The cells were then cycled at C/20 for a single cycle before repeating the rate series in order to separate degradation of the electrode kinetics form the degradation of the electrode capacity. Figure 8a shows how the charge capacity of the electrodes develops during three full rate series. The silicon electrode starts out at a high capacity (~3,200 mAh/g) at C/20 which is retained to approximately 1C. As the rate increases further the capacity rapidly decays to approximately 34% (~1,100 mAh/g) of the starting capacity at 16C. The a-SiN_0.89_ film, while starting at a lower capacity (~1,170 mAh/g) at C/20, shows better rate capabilities, and has capacity retention of approximately 57% (~670 mAh/g) at 16C. This is reflected in colour maps in Fig. 8b, which show the differential capacity analysis of the charge and discharge cycles of the two electrodes, respectively. These figures clearly show that the Si electrode experiences a considerably larger peak shift than the a-SiN_0.89_, both during charge and discharge. The main cause of capacity loss is therefore a shift of the primary lithiation peaks to below the cut-off voltage. Interestingly, the M#d1 peak, which is only seen in the nitride, shifts considerably more than the Si#d1 and Si#d2 peaks, indicating limited kinetics of the matrix lithiation.

During the next two rate series, the capacity of the Si electrode at C/20 is only modestly reduced from ~3,200 mAh/g to approximately 3,110 mAh/g and 2,970 mAh/g, corresponding to 97% and 93% of the initial capacity at C/20, respectively. The capacity at 16C, on the other hand, drops from ~1,100 mAh/g to only 60% and 55% of the initial capacity at 16C; that is, to 660 mAh/g and 600 mAh/g. Given the larger reduction in capacity at high rate compared to that at low rate, the former cannot be attributed to material loss, but rather kinetic limitations which develop in the electrode during cycling. A possible reason for this is silicon’s inability to form a stable SEI layer, which then re-forms every cycle, thus becoming thicker and less penetrable for lithium ions. The a-SiN_0.89,_ in contrast, experienced a small *increase* in capacity at all rates in the subsequent series. The capacity at C/20 in the second and third series increased to 1,190 mAh/g and 1,210 mAh/g, respectively, corresponding to 103% and 104% of the initial C/20 capacity, while the capacity at 16C increased from 670 mAh/g to 710 mAh/g and 715 mAh/g, respectively; that is, 106% and 107% of the initial capacity at 16C. Contrary to the silicon electrode, the high rate capacity increased more than the low rate capacity, indicating that the electrode kinetics is improved during cycling. This is expected to be related to the maturation of the material as discussed previously, which in itself presents a possible explanation for the increase in capacity seen for the electrodes during the first 100 cycles of long term cycling (Figure 4). It also indicates that, even though silicon is believed to be the main active part of a-SiN_x_ electrodes, the inability of silicon to form a stable SEI is not necessarily inherited by the nitrides. The main reason for this is presumed to be the reduced volumetric change of the nitride compared to pure silicon during cycling, in addition to the matrix phase acting as a stable intermediary layer between the silicon and the SEI.

## Conclusions

In this work we aimed to explore the effects of varying the nitrogen content in amorphous silicon nitride on its electrochemical performance. This was done using ten different thin films, of five different compositions ranging from pure silicon to SiN_0.89_ and two different thicknesses, nominally 40 nm and 120 nm. We found that increasing the nitrogen content lowers the specific reversible capacity and increases the first cycle irreversible capacity of the material, but at the same time drastically improves the cycling stability of the material. We also found that these effects appear gradually in the composition range that was explored, an important implication of which is that the material can be tailored to exhibit different combinations of properties suitable for different applications. The more nitrogen rich electrodes exhibited excellent cycling stability, with negligible capacity loss over 2,000 cycles, while still retaining a reversible capacity several times that of graphite.

Differential capacity analysis indicated that delamination was the primary mode of degradation for thinner films, while thicker films also showed signs of pulverization effects. We observed that nitrogen contents in the top of the explored range were needed to prevent delamination, while material pulverization was reduced already at lower nitrogen contents. This should be taken into account when optimizing a-SiN_x_ for particle based electrodes, which are primarily dependent on material stability, and thus could be stabilized by less nitrogen than was found to be optimal in this thin film study. The silicon nitride films were also found to perform better than pure silicon during rate testing, with the 41 nm a-SiN_0.89_ electrode retaining 57% of the reversible capacity at 16C, compared to 34% for a 42 nm a-Si reference. This analysis also showed that SEI related problems may be less pronounced in silicon nitride than in pure silicon.

In order to make amorphous SiN_x_ commercially viable, these experiments must be extended to powder based electrodes, and a solution for the low first cycle coulombic efficiency must be found; however, the excellent cycling stability, high capacity and rate capability exhibited by a-SiN_x_ in this work, in combination with its flexibility in terms of tuning these properties, clearly demonstrates its potential as a next generation anode material, and, as such, warrants further research.

## Methods

Thin film electrodes of amorphous silicon nitride were prepared in a clean-room environment using plasma enhanced chemical vapour deposition (PECVD, Oxford Instruments Plasmalab System133) with silane (SiH_4_) and ammonia (NH_3_) as precursors. Films were deposited using a substrate temperature of 400 °C, a plasma power of 40 W and chamber pressure of 300 mTorr. Rolled copper foil was used as substrates, which were rinsed with acetone and ethanol before deposition. Silicon nitride films with a range of compositions were made by changing the ratio of the precursor gases. Ten samples were made, with five different compositions and two different thicknesses, nominally 40 nm and 120 nm.

Spectroscopic ellipsometry (V-VASE® J.A. Woollam Co.) was used to determine the thickness and refractive index of the pristine films. This analysis was conducted on films deposited on polished silicon wafers which were placed in the deposition chamber together with the copper substrates. The refractive index at λ = 630 nm was used to estimate the film composition through a commonly used formula proposed by Bustarret, *et al*.^34^

1 $$\frac{[N]}{[Si]}=\frac{4}{3}\frac{({n}_{Si}-{n}_{film})}{({n}_{film}+{n}_{Si}-2{n}_{S{i}_{3}{N}_{4}})}.$$

Here *n*_*Si*_, ${n}_{S{i}_{3}{N}_{4}}$ and *n*_*film*_ are the refractive indices of amorphous silicon, silicon nitride and the film, respectively. The values used for the refractive indices of silicon and silicon nitride in this calculation are 4.21^50^ and 2.01^51^, respectively. The compositions were also analysed using X-ray Photoelectron Spectroscopy (XPS) in order to evaluate the estimates obtained from ellipsometry, as well as to investigate the bonding states of Si and N in the material. This analysis was conducted on a Kratos Axis Ultra DLD spectroscope using monochromated Al Kα X-rays (hν = 1,486.6 eV). Any surface contamination was removed by argon sputtering at 2 kV and 100 µA for 2 minutes before characterization. Peak fitting of XPS spectra was done using a procedure from Ingo, *et al*.^39^. Elemental quantification was done based on O 1s, N 1s, C 1s and Si 2p spectra after Shirley background subtraction^52^. Additionally, quantification was also conducted based on the component distributions of the Si 2p spectra, as described in section 2.1 and Fig. 3. The agreement between these methods was found to be excellent, despite their inherent differences, which adds credibility to the result. Further details of this analysis can be found in the supplementary information.

The surface morphologies of the films were characterized using scanning electron microscopy (SEM, Hitachi TM3000 and JEOL JIB-4500) and optical microscopy. The film thickness, roughness and coverage were analysed using transmission electron microscopy (TEM), which was also used for structural characterization of the film, as well as to examine the substrate-film interface. This analysis was performed in a probe corrected FEI Titan G2 60–300, equipped with an FEI SuperX energy dispersive spectrometer (EDS) and a Gatan GIF Quantum 965 electron energy loss spectrometer (EELS), and operated in both projection and scanning mode (TEM and STEM) at an acceleration voltage of 300 kV.

The film mass densities were determined from the characteristic bulk plasmon energy, E_p_, of the material, as measured by EELS in the TEM. The plasmon energy is extracted from the EELS spectra using the equation^53^

2 $${E}_{p,bound}=\sqrt{{{E}_{max}}^{2}+\frac{{\rm{\Delta }}{{E}_{p}}^{2}}{2}}.$$

Here *E*_*max*_ is the energy of the observed maximum and Δ*E*_*p*_ is the full width at half maximum of the plasmon peak. In a free electron model the plasmon energy can be related to the valence electron density by

3 $${E}_{p}=\hslash \sqrt{\frac{n{e}^{2}}{m{{\epsilon }}_{0}}}.$$

Here E_p_ is the bulk plasmon energy, $\hslash$ is the reduced Planck constant, *n* is the valence electron density, *e* is the elementary charge, *m* is the electron mass and ${{\epsilon }}_{0}$ is the permittivity of vacuum. Since the electrons in the material in question are not necessarily free, a correction of the plasmon energy is made by using the band gap of the material as a measure of the bonding energy of the electrons as proposed by Egerton^53^

4 $${E}_{p}=\sqrt{{{E}_{p,bound}}^{2}-{{E}_{g}}^{2}}.$$

Here *E*_*p*_ and *E*_*p*,*bound*_ are the plasmon energies in systems with the same valence electron density with free and bound electrons, respectively, and *E*_*g*_ is the band gap of the material. Given the material’s composition, the mass density can then be calculated by

5 $${\rho }_{p}=n\,\ast \,\frac{{\sum }_{x=(Si,N,O,H)}\,{f}_{X}{M}_{X}}{{N}_{A}\,{\sum }_{x=(Si,N,O,H)}\,{f}_{X}{v}_{X}}.$$

Here *N*_*A*_ is the Avogadro constant, and *f*_*X*_, *M*_*X*_ and *v*_*x*_ are the molar fraction, molar mass, and number of valence electrodes per atom of element X (Si, N, O, H). The atomic fraction of hydrogen was estimated to be approximately 0.2 by secondary ion mass spectrometry (SIMS), and the remaining fractions were taken from XPS measurements. For reference, this analysis was conducted on a pure c-Si and c-Si_3_N_4_, which are of known density. The film densities were also estimated by linear interpolation between the density of amorphous silicon (2.2 g/cm^3^ ^54^) and amorphous SiN_1.31_ (2.9 g/cm^3^ ^55^) based on the composition, *x* = [N]/[Si], by the relationship

6 $${\rho }_{l}={\rho }_{Si}+({\rho }_{Si{N}_{1.31}}-{\rho }_{Si})\frac{x}{1.31}.$$

Electrochemical testing of the films was conducted in 2032 coin cells against a lithium metal counter electrode. Cells were assembled in an argon filled glove box with a Celgard 3401 separator and an electrolyte consisting of 1 M LiPF_6_ dissolved in a 1:1:3 mixture by mass of ethylene carbonate (EC)/propylene carbonate (PC)/dimethyl carbonate (DMC), with 1 wt.% vinylene carbonate (VC) and 5 wt.% fluoroethylene carbonate (FEC). Galvanostatic cycling was performed between 0.05 V and 1 V vs. Li/Li^+^ using an Arbin BT-2000 galvanostat/potentiostat. These cells were started with a formation procedure consisting of 6 cycles at a rate of C/20 in order to allow the expected conversion reaction to finish, after which regular cycling was continued at 1C. These tests were run on films of all compositions and thicknesses. Some electrodes were also subjected to rate testing with current rates increasing from C/4 to 16C, doubling the rate by every 5 cycles. The C-rates for these experiments were estimated from the compositions by linear interpolation between 1,200 mAh/g for SiN_0.89_^33^ and 3,579 mAh/g for Si, as described in the supplementary information. When compared to the charge capacity obtained in the experiments during the initial formation cycles, these estimates were found to be satisfactory.

## Electronic supplementary material

Supplementary information
