# Supplementary material for: Substoichiometric Silicon Nitride – An Anode Material for Li-ion Batteries Promising High Stability and High Capacity
Source: Sci Rep. 2018 Jun 5;8:8634. doi: 10.1038/s41598-018-26769-8 (PMC5988813; doi:10.1038/s41598-018-26769-8)
Supplement: Supplementary file 1 — Supplementary information [file 41598_2018_26769_MOESM1_ESM.pdf]

Supplementary information to the paper:

Substoichiometric Silicon Nitride – An Anode Material for Li-ion Batteries Promising High Stability and High Capacity

Asbjørn Ulvestad[a*], Hanne F. Andersen[a], Ingvild J. T. Jensen[c], Trygve T. Mongstad[a], Jan Petter Mæhlen[a], Øystein Prytz[b], and Martin Kirkengen[a]

[a] Department of Battery Technology, Institute for Energy Technology
P.O. Box 40, NO-2027 Kjeller, Norway

[b] Department of Physics, Centre for Materials Science and Nanotechnology, University of Oslo
P.O. Box 1048 Blindern, NO-0316 Oslo, Norway

[c] SINTEF Industry
P.O. Box 124 Blindern, NO-0314 Oslo, Norway

*Corresponding Author:
Asbjørn Ulvestad
Institute for Energy Technology
P.O. Box 40
NO-2027 Kjeller, Norway
Tel.: +47 47 288 366
Fax: +47 63 816 356
asbjorn.ulvestad@ife.no

1 Thin film deposition

Thin films were deposited using plasma enhanced chemical vapor deposition (PECVD, Oxford Instruments Plasmalab System133) using silane and ammonia as precursors. All films were deposited using a substrate temperature of 400 °C, a plasma power of 40 W and a chamber pressure of 300 mTorr, onto two different substrates simultaneously: rolled copper foil (for electrochemical testing) and a polished silicon wafer (for ellipsometry). The parameters used for the deposition of the different films can be seen in Supplementary Table 1.

Supplementary Table 1: Parameters used in the PECVD deposition of the different thin films.

Film index	Substrate temperature [C]	Chamber pressure [mTorr]	Flow rate, Q [sccm]		Deposition time [s]
			Silane (SiH_4)	Ammonia (NH_3)	
A1	400	300	25	0	60
A2	400	300	25	0	180
B1	400	300	25	10	60
B2	400	300	25	10	180
C1	400	300	25	20	60
C2	400	300	25	20	180
D1	400	300	25	30	60
D2	400	300	25	30	180
E1	400	300	25	40	60
E2	400	300	25	40	180

2 X-ray Photoelectron Spectroscopy

2.1 Si 2p peak fitting

The relative distribution of silicon atoms in Si-Si_4 (pure Si), Si-Si_3N (Si_3N), Si-Si_2N_2 (Si_3N_2), Si-SiN_3 (Si_3N_3), and Si-N_4 (Si_3N_4) configurations was determined by fitting Voigt functions representing each of these configurations to the Si 2p peak using a procedure by Ingo, et al. [38]. This fitting performed on the spectra obtained from the different thin films can be seen in Supplementary Figures 1 to 6.

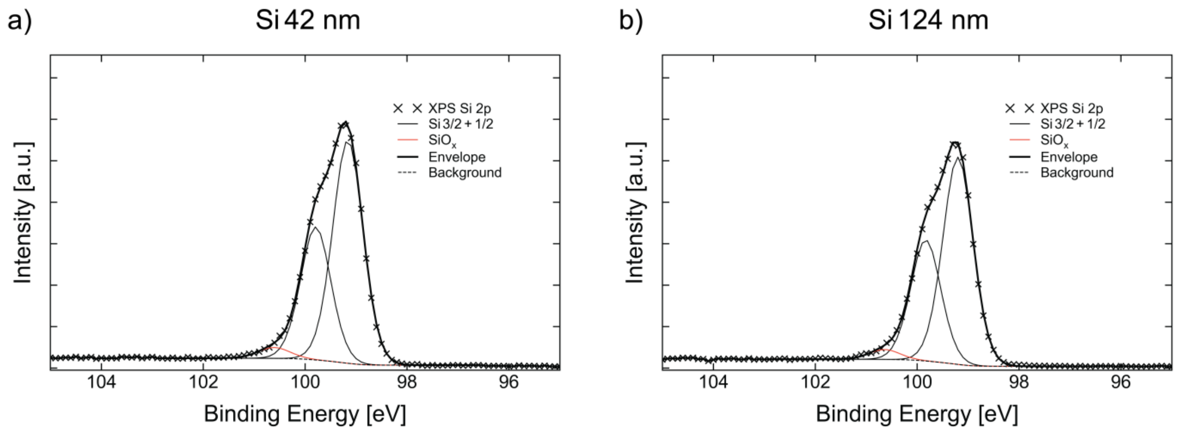

Supplementary Figure 1: Fitting of Voigt functions to Si 2p core level XPS spectra obtained from the 42 nm and 124 nm Si thin films.

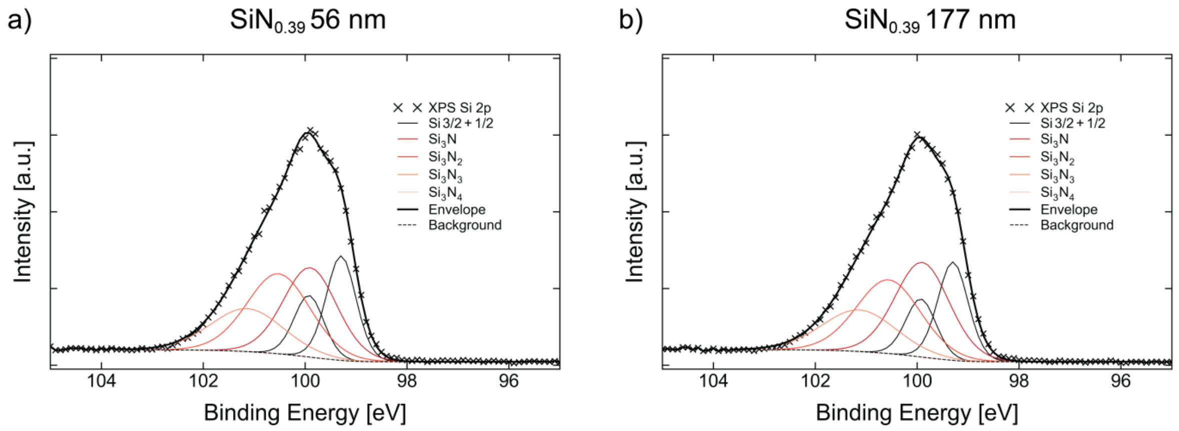

Supplementary Figure 2: Fitting of Voigt functions to Si 2p core level XPS spectra obtained from the 56 nm and 177 nm $SiN_{0.39}$ thin films.

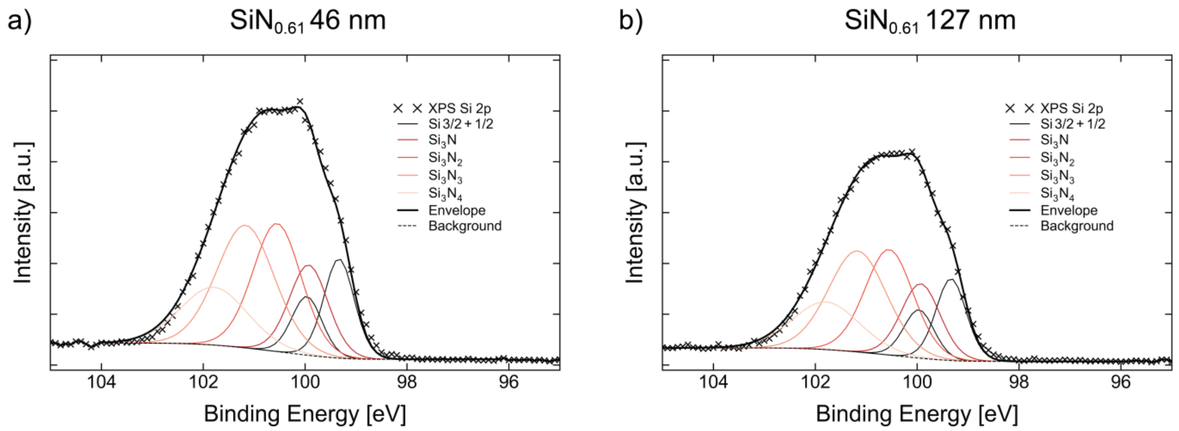

Supplementary Figure 3: Fitting of Voigt functions to Si 2p core level XPS spectra obtained from the 46 nm and 127 nm $SiN_{0.61}$ thin films.

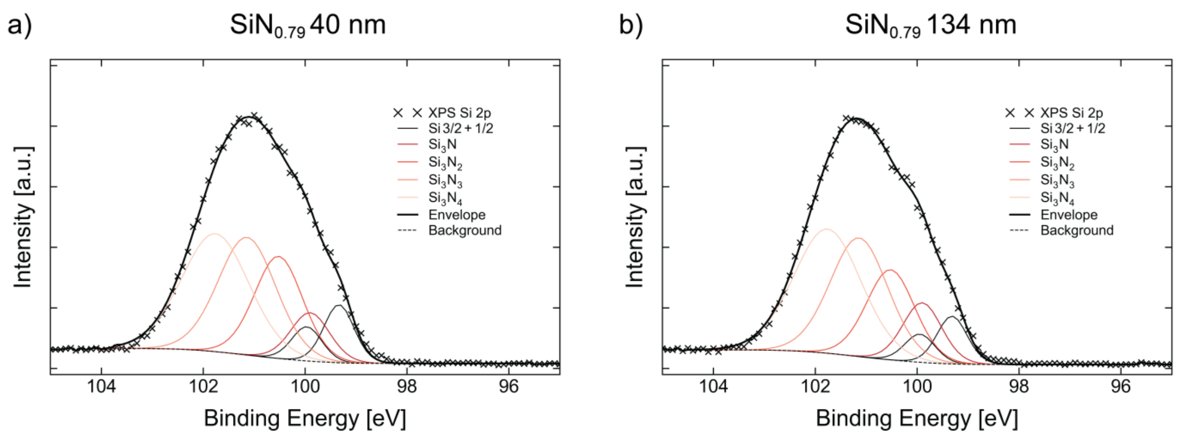

Supplementary Figure 4: Fitting of Voigt functions to Si 2p core level XPS spectra obtained from the 40 nm and 134 nm $SiN_{0.79}$ thin films.

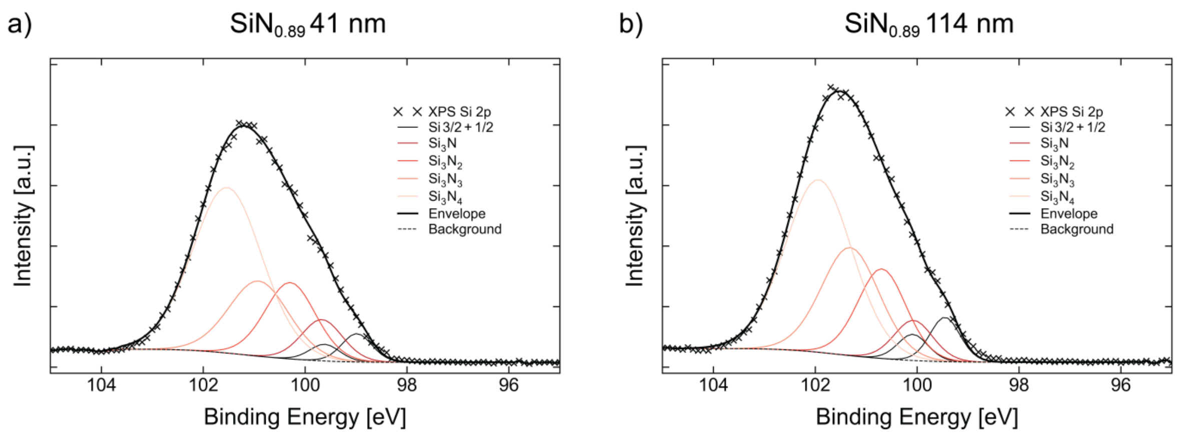

Supplementary Figure 5: Fitting of Voigt functions to Si 2p core level XPS spectra obtained from the 41 nm and 114 nm $SiN_{0.89}$ thin films.

2.2 Quantification

The quantification the nitrogen to silicon ratio of the films from the XPS data was conducted using the common method of comparing the intensities of N 1s and Si 2p peaks of each sample after Shirley background subtraction[52], scaled by relative sensitivity factors (RSFs). The accuracy of this quantification necessarily depends on accurately determined RSFs; hence to check the validity of the obtained compositions, another method was also employed, which calculates the N content of the films using the fitted SiN_x components in the Si 2p spectrum. The fitting procedure for determining this distribution is detailed in the main paper, and the resulting curve fits can be found in in the previous section. A comparison of the results from these methods can be seen in Supplementary Table 2, showing that the compositions are within approximately 2 at.% for all samples. The good agreement between the two methods despite their inherent differences, adds credibility to the reliability of the result. As the fitting was done without any restrictions on the relative area of the SiN_x components, the excellent agreement between the two quantification methods regarding the N content also strengthens the credibility of the Si 2p analysis above.

Supplementary Table 2: Results from the different methods for quantifying the nitrogen to silicon ratio of the thin films, and their difference. Method 1 is based on the scaled relative intensity of the N 1s and Si 2p peaks, while method 2 is based on the component distribution extracted from Si 2p as detailed in the main paper and shown in the previous section.

Film index	Quantification method 1		Quantification method 2		Difference	
	x = [N]/[Si]	at.% N	x	at.% N	Δx	Δ at.% N
A1	0,022	2.1	0,000	0.0	-0,022	-2.1
A2	0,021	2.0	0,000	0.0	-0,021	-2.0
B1	0,380	27.5	0,360	26.4	-0,020	-1.1
B2	0,402	28.7	0,359	26.4	-0,042	-2.2
C1	0,587	37.0	0,590	37.1	0,002	0.1
C2	0,627	38.5	0,588	37.0	-0,039	-1.5
D1	0,794	44.3	0,791	44.2	-0,004	-0.1
D2	0,780	43.8	0,824	45.2	0,043	1.3
E1	0,912	47.7	0,950	48.7	0,038	1.0
E2	0,873	46.6	0,902	47.4	0,029	0.8

3 Density determination

3.1 Bulk plasmon energy determination from electron energy loss spectroscopy (EELS)

The bulk plasmon energy of each film was determined using EELS analysis. This analysis was conducted in an FEI Titan G2 60-300 TEM operating at 300 kV on cross-section TEM samples prepared using a JEOL JIB-4500 focused ion beam system. Spectra were aquired using a Gatan GIF Quantum 965 EELS spectrometer. For each sample, several hundred spectra were aquired over the thickness of the film, aligned and summed, resulting in a raw spectrum as expemplified in Supplementary Figure 6 by the spectrum recorded from the 114 nm $SiN_{0.89}$ thin film. The spectra were deconvoluted using the Fourier-log method in order to remove plural scattering contributions, also seen an example of in the same figure. The resulting deconvoluted spectra were used to extract the energy at the bulk plasmon peak maximum and the peaks full width at half maximum (FWHM). Using these values and the procedure outlined in the main paper, the bulk plasmon energy was calculated. The parameters used in this analysis and resulting plasmon energies for the different samples can be seen in Supplementary Table 3.

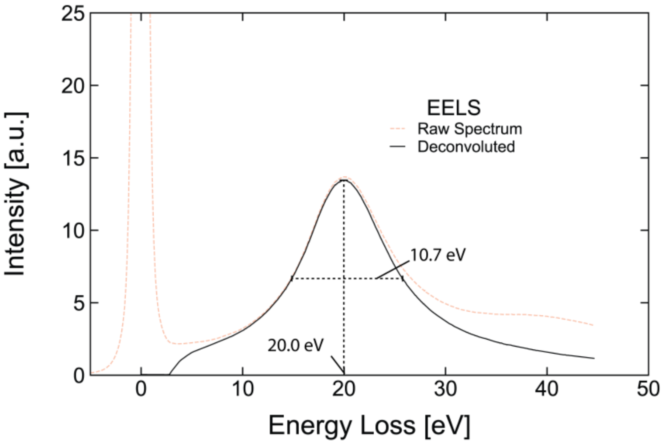

Supplementary Figure 6: EELS spectrum acquired from the 114 nm $SiN_{0.89}$ thin film, both as-recorded and deconvoluted using the Fourier-log method. The position of the bulk plasmon peak maximum and FWHM are indicated.

Supplementary Table 3: The bulk plasmon peak maximum position and full width at half maximum (FWHM) extracted from deconvoluted EELS spectra collected from the different thin films and two references (c-Si and c-Si_3N_4), as well as the bulk plasmon energy calculated from these measurements.

	Films					References	
**Samples**	**Si**	**$SiN_{0.39}$**	**$SiN_{0.61}$**	**$SiN_{0.79}$**	**$SiN_{0.89}$**	**c-Si**	**c-Si_3N_4**
Bulk plasmon peak maximum energy [eV]	16.7	17.7	18.8	18.9	20.0	16.4	23.0
Bulk plasmon peak FWHM [eV]	4.9	8.3	8.7	11.5	10.7	5.2	14.8
Bound bulk plasmon energy (Eq. 2) [eV]	17.0	18.6	19.8	20.6	21.4	16.8	25.3

3.2 Calculation of mass density from bulk plasmon energy

The densities of the films are determined from the bulk plasmon energies in the manner outlined in the methods section in the main paper. A correction is first made in order to obtain approximate free electron environment equivalents of the bulk plasmon energies from the measured *bound* bulk plasmon energies by using equation (4) and the band gap of the materials. These values are then used to estimate the valence electron densities using equation (3), which are then related to the mass density of the material through equation (5). In the latter equation, the hydrogen content of the film is assumed to be 20 at. % based on an estimate obtained from secondary ion mass spectrometry (SIMS) analysis of the 114 nm $SiN_{0.89}$ film. The contents of silicon, nitrogen and oxygen in the thin films were otherwise determined by XPS. The result of this analysis, as well as an overview of the parameters used, is shown in Supplementary Table 4.

Supplementary Table 4: Overview of the parameters used to determine the mass density of the material from the bulk plasmon energy, band gap and composition of the material different thin films, as well as from c-Si and c-Si_3N_4 references.

Composition	Films					References	
	**Si**	**$SiN_{0.39}$**	**$SiN_{0.61}$**	**$SiN_{0.79}$**	**$SiN_{0.89}$**	**c-Si**	**c-Si_3N_4**
Bound bulk plasmon energy from EELS [eV]	17.0	18.6	19.8	20.6	21.4	16.8	25.3
Assumed band gap* [eV]	1.90	2.00	2.15	2.40	2.65	1.12	5.00
Free bulk plasmon energy (Eq. 4) [eV]	16.9	18.5	19.7	20.4	21.2	16.6	24.8
Valence electron density (Eq. 3) [10^{29} m^{-3}]	2.08	2.49	2.81	3.02	3.26	1.99	4.38
Mass density (Eq. 5) [g/cm^3]	2.18	2.15	2.25	2.31	2.44	2.38	3.24

*The band gap values used for the thin films are interpolated from Guraya et al. (1990) [38], while the band gap values for the c-Si and c-Si_3N_4 references are from Bludau et al. (1974) [36] and Sze and Ng (2006) [37], respectively.

4 Electrochemical cycling parameters

4.1 Rate determination

The conversion reaction of silicon nitrides has not been conclusively determined, and the materials' theoretical capacity therefore cannot be directly calculated. Suitable specific capacity values to use in determining the C-rates have therefore been estimated based on the previously reported value of 1,200 mAh/g [33] for $SiN_{0.89}$ and the theoretical capacity of 3,579 mAh/g for silicon. In order to limit the time the films were stored between deposition and cell production, thorough characterization of the films was not performed until after electrochemical testing started; hence the capacity estimates were made by linear interpolation based on the relative amount of nitrogen to silicon in the deposition plasma as a crude measure of the nitrogen content of the deposited films. The film thickness, density and the related mass loading were similarly unknown. The film thicknesses were therefore assumed to be equal to the target values of 40 and 120 nm, while a rough estimate of the density was simply taken as the average of silicon (2.33 g/cm^3) and silicon nitride (3.2 g/cm^3) [35], 2.8 g/cm^3, for all samples, resulting in estimated areal loadings of 0.011 and 0.034 mg/cm^2 for the 40 nm thick films and the 120 nm thick films, respectively. Note that this rough estimate of the active mass was only used to estimate suitable current rates. For all other matters, measured values were used, determined from film thicknesses measured using ellipsometry, and densities determined from the bulk plasmon energy as measured in electron energy loss spectroscopy through the procedure detailed in the methods section of the main paper. An overview of the estimated values can be seen in Supplementary Table 5, together with the actual mass loading. The latter was used in the determination of the actual specific capacity of the materials. The currents used during cycling of the different films, calculated based on the information in Supplementary Table 5, can be seen in Supplementary Table 6. The estimates for the mass were found to be adequate for all the samples and generally slightly higher than expected, with the exception of the films with composition $SiN_{0.39}$.

Supplementary Table 5: Overview of the estimated specific capacity and mass loading of the films, used in determining the current rates used during cycle testing, and the actual mass loading used in the determination of the specific capacity of the materials.

Film index	Estimated specific capacity [mAh/g]	Estimated mass loading [mg/cm^2]	Actual mass loading [mg/cm^2]
A1	1,200	0.011	0.009
A2	1,200	0.034	0.027
B1	1,795	0.011	0.012
B2	1,795	0.034	0.038
C1	2,390	0.011	0.010
C3	2,390	0.034	0.029
D1	2,985	0.011	0.009
D2	2,985	0.034	0.031
E1	3,579	0.011	0.010
E2	3,579	0.034	0.028

Supplementary Table 6: Overview of the current densities used during cycling of the different films at different C-rates, and the absolute currents used for cycling of ⌀15 mm electrodes of each film.

Film index	Assumed capacity of a ⌀15 mm electrode [µAh]	Current rate for a ⌀15 mm electrode [µA]			Assumed areal capacity [µAh/cm2]	Current density [µA/cm^2]		
		C/20	C/3	1C		C/20	C/3	1C
A1	23,3	1,17	7,78	23,33	13,2	0,66	4,40	13,20
A2	72,1	3,60	24,03	72,10	40,8	2,04	13,60	40,80
B1	34,9	1,74	11,63	34,89	19,7	0,99	6,58	19,75
B2	107,8	5,39	35,95	107,85	61,0	3,05	20,34	61,03
C1	46,5	2,32	15,49	46,46	26,3	1,31	8,76	26,29
C3	143,6	7,18	47,87	143,60	81,3	4,06	27,09	81,26
D1	58,0	2,90	19,34	58,02	32,8	1,64	10,95	32,84
D2	179,3	8,97	59,78	179,35	101,5	5,07	33,83	101,49
E1	69,6	3,48	23,19	69,57	39,4	1,97	13,12	39,37
E2	215,0	10,75	71,68	215,04	121,7	6,08	40,56	121,69

4.2 Galvanostatic voltage-capacity curves

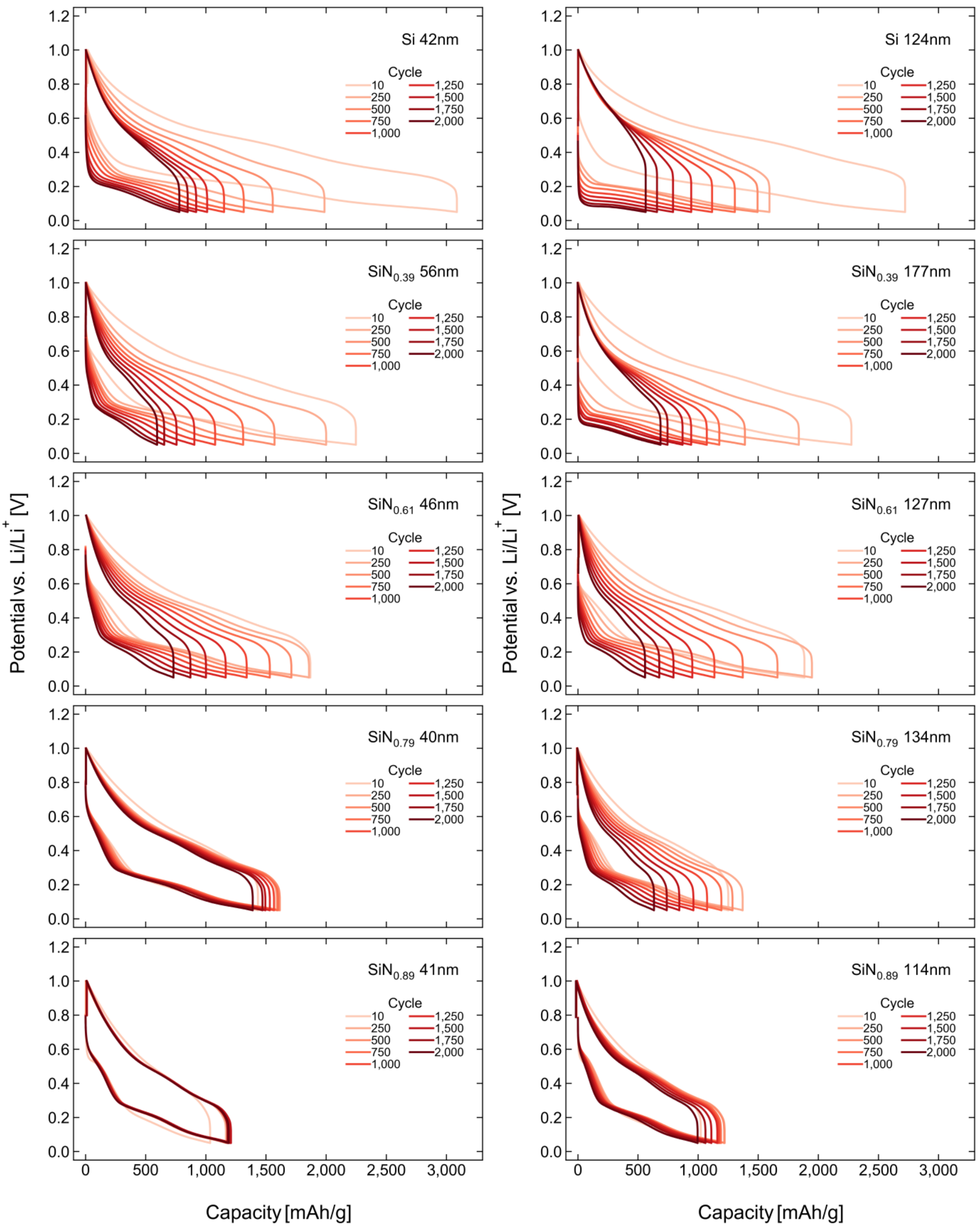

Supplementary Figure 7: Galvanostatic voltage-capacity curves from cycles 10, 250, 500, 750, 1000, 1250, 1500, 1750 and 2000 of the ten different thin films, cycled at 1C.
